# Derivation of Ethnically Diverse Human Induced Pluripotent Stem Cell Lines

**DOI:** 10.1038/srep15234

**Published:** 2015-10-20

**Authors:** Eun Ah Chang, Martin L. Tomov, Steven T. Suhr, Jiesi Luo, Zachary T. Olmsted, Janet L. Paluh, Jose Cibelli

**Affiliations:** 1 Michigan State University, Departments of Animal Science and Physiology, East Lansing, MI, 48824; 2 State University of New York Polytechnic Institute, Colleges of Nanoscale Science and Engineering (SUNY PI CNSE), Nanobioscience, Albany, NY, 12203; 3 LARCEL, Laboratorio Andaluz de Reprogramación Celular, BIONAND, Andalucía, 29590, Spain.

## Abstract

The human genome with all its ethnic variations contributes to differences in human development, aging, disease, repair, and response to medical treatments and is an exciting area of research and clinical study. The availability of well-characterized ethnically diverse stem cell lines is limited and has not kept pace with other advances in stem cell research. Here we derived xenofree ethnically diverse-human induced pluripotent stem cell (ED-iPSC) lines from fibroblasts obtained from individuals of African American, Hispanic-Latino, Asian, and Caucasian ethnic origin and have characterized the lines under a uniform platform for comparative analysis. Derived ED-iPSC lines are low passage number and evaluated *in vivo* by teratoma formation and *in vitro* by high throughput microarray analysis of EB formation and early differentiation for tri-lineage commitment to endoderm, ectoderm and mesoderm. These new xenofree ED-iPSC lines represent a well-characterized valuable resource with potential for use in future research in drug discovery or clinical investigations.

The promise of stem cells to recapitulate aspects of normal or impaired development, mature cellular function and disease states provides hope for new insights applicable to regenerative medicine, cell and tissue therapies, and drug studies in human health care. Stem cell research is already providing important information on monogenetic and metabolic diseases by utilizing patient iPSCs for disease mechanism studies surrounding the affected cell types^1^,^2^,^3^. Analysis of early or late onset monogenetic diseases includes neurodegeneration, such as the role of SMN1 in early motor neuron death in a spinal muscular atrophy patient^4^ or late onset familial Parkinson’s disease that is exacerbated by mutation of LRRK2 in dopaminergic neurons of disease patients^5^,^6^. Disorder of carbohydrate metabolism in type I diabetes with insufficient production of insulin hormone may also be approachable through stem cell therapies^7^. By expanding the availability of iPSC lines that reflect age, gender or ethnic group further refinement in understanding phenotypically different responses to disease onset or drug treatments versus healthy controls is expected.

Here we present details of the derivation and characterization of new high quality ethnically diverse-induced pluripotent stem cells (ED-iPSC) lines of low passage number and of African American, Hispanic-Latino, Caucasian, and Asian ethnicity. Derived ED-iPSC lines have undergone core quality control to be free of mycoplasma, endotoxins, bacteria, yeast, mold and viruses and analyzed for normal karyotype. The ED-iPSC lines were maintained *in vitro* on either human foreskin fibroblasts (HFF) or on feeder-free extracellular xenofree matrix. Pluripotency analysis was done by teratoma formation *in vivo* as well as evaluation of embryoid body (EB) formation and subsequent tri-lineage commitment to early differentiation *in vitro*. Comparative *in vitro* analysis was optimized by use of high throughput custom lithography templated microarrays to generate uniformly sized EBs for validating mulit-lineage commitment^8^. These xenofree ED-iPSC lines are expected to be an important new resource for stem cell research to allow investigation into ethnic origin contributions for biomedical discovery towards clinical applications.

## Results

### Derivation of human ED-iPSC lines from African American, Hispanic-Latino, Caucasian, and Asian ethnicities

**Figure 1:**
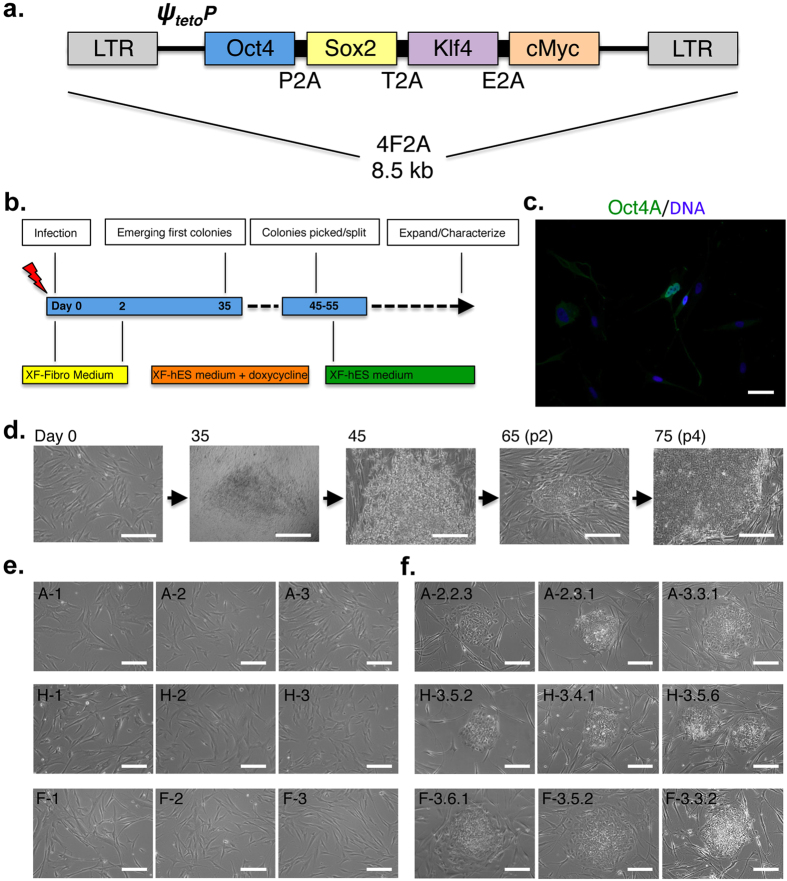
Derivation of ethnically diverse (ED)-iPSC lines from primary human fibroblasts. (**a**) Addgene plasmid 20321 (TetO-FUW-OSKM and 20342; FUW-M2rtTA) is a single polycistronic vector using four-factor 2A (4F2A) doxycycline (DOX)-inducible lentivirus encoding mouse cDNAs for Oct4, Sox2, Klf4 and cMyc separated by three different 2 A peptides (P2A, T2A, and E2A, respectively; Carey *et al.*, 2009). (**b**) Schematic of the process of reprogramming and culture conditions. (**c**) 48-hours after infection, less than 15% of fibroblasts were immunopositive for viral-derived Oct4 by immunocytology (scale bar is 50 μm). (**d**,**f**) After 35 days, infected ED fibroblasts generated stem cell-like colonies that were isolated for derivation of ED-iPSC lines. (**e**) Original cell morphology of parental ED-fibroblasts. Images in (**d**–**f**) are phase contrast (scale bars are 200 μm).

In evaluating genetic contributions to human disease, the generation of induced pluripotent stem cells (iPSCs) constitutes one of the most exciting scientific breakthroughs in the last 20 years. Although multiple sources of pluripotent Caucasian lines exist, there is limited availability of ethnically diverse (ED) iPSC lines. To provide a new high quality resource of ED-iPSCs for biomedical research, we obtained primary human fibroblasts from African American (AA), Hispanic-Latino (HL) and Asian (AS) origins as well as Caucasian parental lines (Coriell Institute, Camden, NJ) and used these to derive ED-iPSC lines (Table 1). To reprogram fibroblasts, polycistronic lentiviral plasmid vectors with tet-inducible expression TetO-FUW-OSKM and FUW-M2rtTA (9; plasmids 20321 and 20342, Addgene) were used and fibroblasts positive for viral derived Oct4 identified by immunocytology (Fig. 1a–c). The polycistronic cassette encodes four factors that are Oct4, Sox2, Klf4, and c-Myc mouse genes whose expression was tracked by semi-quantitative RT-PCR. Over a time course of day 35 to 75, cells grown in 2D culture under xenofree conditions on human foreskin fibroblasts (HFF) were monitored for the appearance of stem cell like clustered colonies (Fig. 1d–f). Derived stem cell lines were maintained on HFF and standard quality control tests done to ensure safety of the iPSCs in regard to absence of contaminating mycoplasma or other biological agents. Karyotyping on G-banded metaphases of the iPSCs was outsourced (Table 2, n = 20 cells per iPSC line; Cell Line Genetics; Madison, WI). Fourteen of the 17 ED-iPSC lines displayed normal karyotyping by this method and were further analyzed. The remaining lines were stored and may be useful for analysis of phenotypes associated with specific chromosomal regions.

### Analysis of ED-iPSC lines for pluripotency by genetic and cell biological biomarker profiles and teratoma analysis

**Figure 2:**
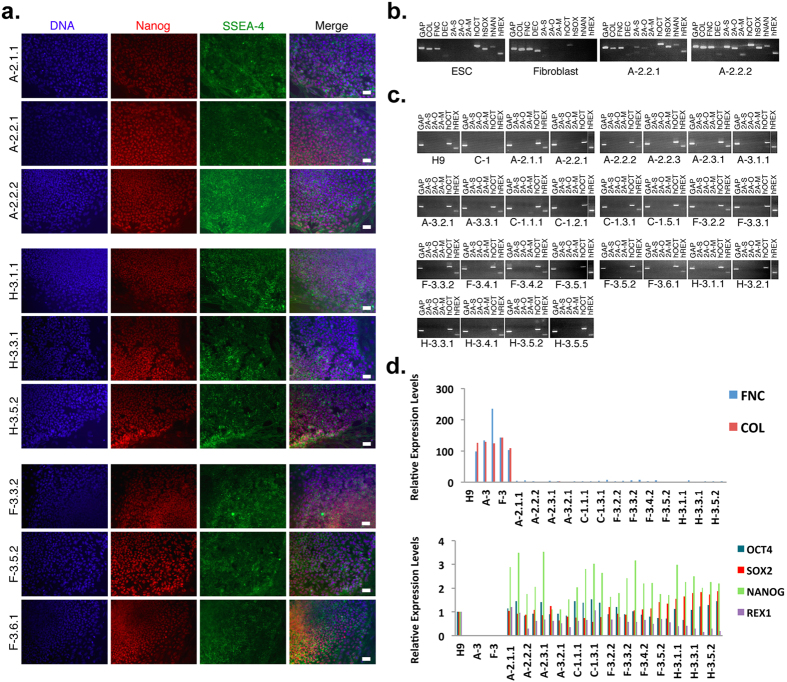
Characterization of gene expression in reprogrammed ethnically diverse (ED)-iPSC lines. (**a**) Representative immunocytology biomarker analysis of pluripotency in 9 ED-iPSC lines. DNA is in blue by Hoechst, Nanog is in red and SSEA-4 is in green (scale bar is 50 μm). A consistent exposure time was used for all images. The variation in intensity is expected due to different cell concentrations. (**b**) Pluripotency-related genes are expressed from the endogenous loci in 26 ED-iPSC lines, while the virally delivered transgene is predominantly silenced revealed by semi-quantitative RT-PCR. Primers were validated in H9 ESCs, parental fibroblasts (A-2), and two of iPSC lines (A-2.2.1 & A-2.2.2); collagen (COL), fibrinonectin (FNC), descortin (DEC), transgene; 2 A-S(Sox2), 2 A-O(Oct4), and 2 A-M(C-myc) and endogeneous gene; hOCT (Oct4), hSOX (Sox2), hNAG (Nanog) and hREX (human REX). (**c**) Semi-quantitative expression of the silenced three transgenes and expressed two endogenous pluripotency genes shown in 26 iPSC lines. GAP (GAPDH) is used as a loading control for each lane. (**d**) Expression of fibronectin (FNC) and collagen type 1α2 (COL) in individual parental fibroblasts (A-2, A-3, C-1, F-3, and H-3) by quantitative RT-PCR. No FNC or COL expression is observed in the ED- iPSCs or H9 hESCs. Expression for OCT4, SOX2, NANOG, and REX1 levels is increased in ED-iPSCs relative to H9 hESCs. PCR reactions were normalized against internal controls (GAPDH) and plotted relative to expression levels in H9 hESCs.

**Figure 3:**
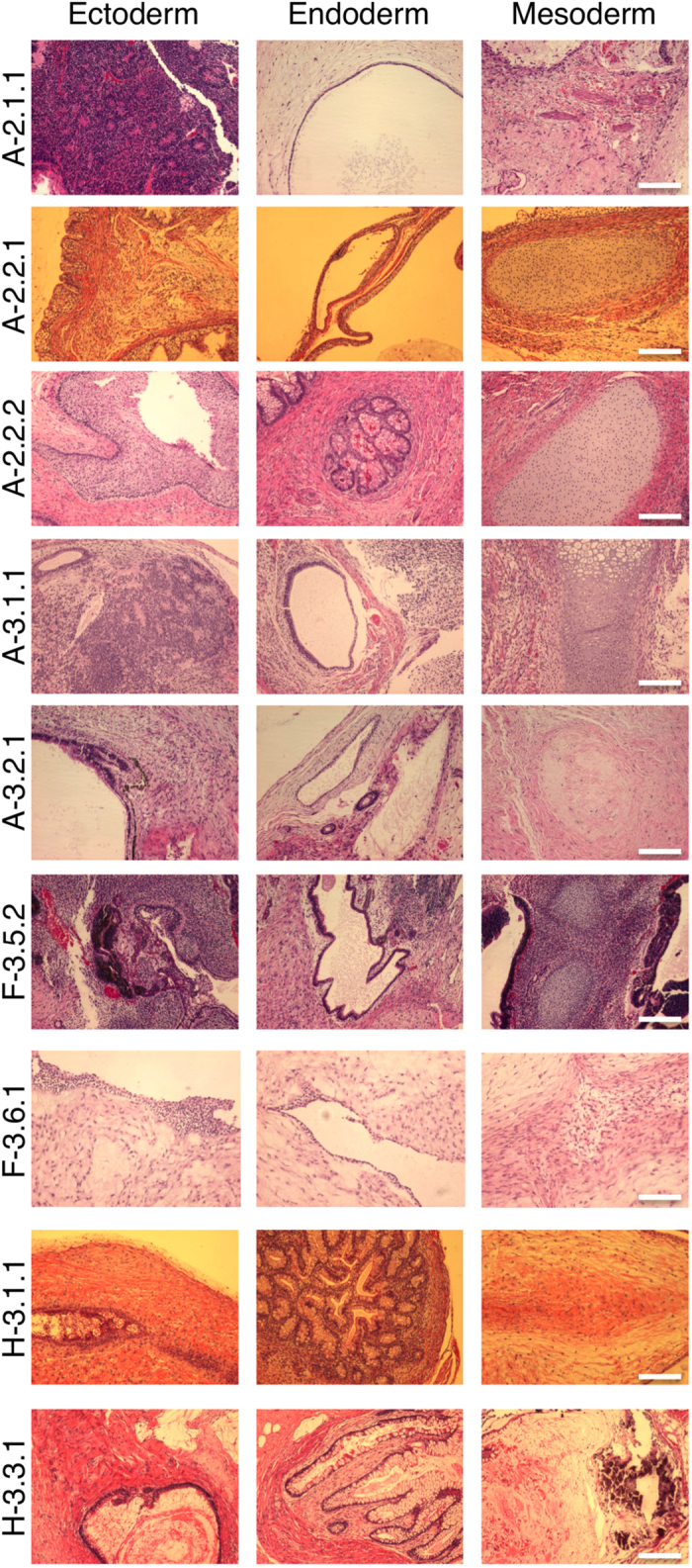
Teratoma formation by ED-iPSCs in Nod scid gamma (NSG) mice. (**a**) Representative image series of hematoxylin-eosin (H-E) stained sections from a formalin-fixed teratomas produced from nine of the ED-iPSC lines representing Asian, African American and Hispanic-Latino ethnicity. Each line formed mature, cystic teratomas with tissues representing the three embryonic germ layers including squamous epithelium, neuroectodermal tissues and melatonin formation (ectoderm), respiratory epithelium and intestinal glandular epithelium (endoderm) and cartilage, bone and connective tissues (mesoderm). Scale bars are 0.2 cm.

The ED-iPSC lines we generated from reprogramming of fibroblasts of African American, Hispanic-Latino, Caucasian and Asian ethnicities were analyzed by a platform of tests that included immunocytology for pluripotency markers Nanog and SSEA-4 (Fig. 2a) and semi-quantitative RT-PCR analysis of gene expression during reprogramming of fibroblasts to pluripotency (Fig. 2b,c). Primers sets for RT-PCR were designed to recognize several classifications of target. These are, Set 1: an internal control housekeeping gene GAPDH (GAP) expressed at similar levels in most cell types and allows normalization of samples. Set 2: genes associated with fibroblast phenotype. Targets included collagen type 1α2 (COL), fibronectin (FNC), and decorin (DEC). Set 3: the lentiviral transgenes that are expressed to induce pluripotency but shut down when iPSCs have fully reprogrammed to pluripotency. To ensure that only mRNAs expressed from the transgenes—and not endogenous genes—were recognized, the three primer sets were designed with one primer recognizing the 2 A elements unique to the viral vector and a second primer targeting the mouse Sox2 (2 A-S), the mouse Oct4(2 A-O), or the mouse cMyc (2 A-M) transgenes. Set 4: Endogenous human genes that should reactivate in iPSCs. The primers selected included human Oct 4 (hOCT), SOX2 (hSOX), nanog (hNAG) and REX1 (hREX). The evaluation of primer sets is shown in (Fig. 2b), agarose gels of PCR products in (Fig. 2c), and quantitative RT-PCR data shown by histogram in (Fig. 2d). The gold standard test of pluripotency is generation of teratomas *in vivo*, as shown in (Fig. 3). Our analysis is consistent with successful reprogramming to generate human iPSCs from ethnically diverse origins. In our study, several lines were derived from each fibroblast source and a subset of these validated by teratoma formation. No obvious differences were observed between the fibroblast resources in regard to the timeline for reprogramming or efficiency, as validated by qRT-PCR, nor in ability of tested lines to derive teratomas. However, we did attempt to derive an iPS line from a Pima Indian fibroblast line, which was unsuccessful after several attempts. The reason for this difficulty is unknown since the fibroblast line was derived from a similar age group as other ethnically diverse fibroblasts used in this study.

### Microarray Embryoid Body formation and tri-lineage commitment of ED-iPSCs *in vitro*

**Figure 4:**
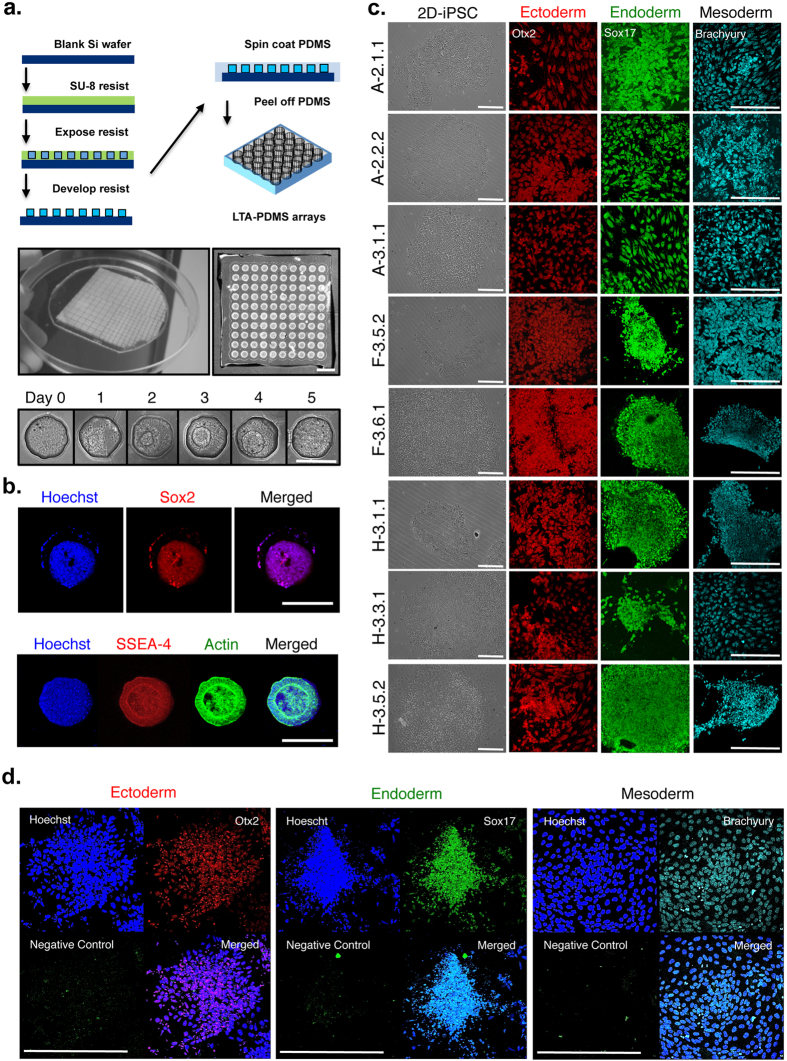
*In vitro* tri-lineage commitment to differentiation by ED-iPSCs. (**a**) Schematic of the process flow to generate LTA-PDMS grids used for high throughput embryoid bodies (EBs) templating (top image), a PDMS mold of arrays prepared on a 4 inch wafer (middle), and 5 day time course of EBs formation in 200 μm LTA-PDMS grid wells (bottom image; (N > 400; scale bar is 200 μm). (**b**) EBs characterization by Hoechst, immunocytology for pluripotency markers Sox2 or SSEA-4, and Actin (phalloidin) staining. The F3.5.2 ED-iPSCs EBs shown was templated in a 200 μm well to day 5. Scale bar is 200 μm. (**c**) Representative images of differentiated 200 μm ED-iPSCs EBs using a Human pluripotent stem cell identification kit. Left column image shows bright field of pluripotent ED-iPSCs 2D colonies. Differentiated ED-iPSCs were fixed for ICC analysis at day 4 for the ectoderm and endoderm lineages and at day 3 for the mesoderm lineage. Antibodies used for immunocytology were indicated at the top of each column. Scale bars are 200 μm. (**d**) Colocalization of Hoescht nuclear stain and germ layer specific markers, listed in each image, to the nucleii of differentiated iPSC cells. Negative control is secondary antibody only. Representative images for germ layer commitment are shown for the iPSC line A2.2.2 at day 4 for ectoderm and endoderm and at day 3 for mesoderm. Scale bars are 200 μm.

The ability of the iPSCs to form embryoid bodies (EBs) and commit to tri-lineage differentiation was compared *in vitro*. For uniform evaluation we generated custom 200 μm microwells by photolithography and coating with poly-dimethylsiloxane (PDMS) (Fig. 4a). The formation of ED-iPSC EBs *in vitro* was done by first dissociating 2D colonies to single cells or by mechanical passaging to generate 2D clusters then loading wells as previously described^8^. EB formation including a characteristic internal cyst occurred with similar efficiency by all lines (Fig. 4b). To evaluate ability of iPSCs to commit to multi-lineage early differentiation, we used a commercially available tri-lineage germ layer commitment kit (RnD Systems, Minneaopolis, MN) with EBs over a five day period then stained cells for biomarkers corresponding to each germ layer that included, Otx2 for ectoderm, Brachyury for mesoderm, and Sox17 for endoderm (Fig. 4c-d). We observed that over 94% of the cells stain positively for the corresponding germ layer marker. Minimal cell death was observed during the differentiation, consistent with high quality pluripotent stem cell lines.

## Discussion

Here we generated high quality xenofree ethnically diverse-human induced pluripotent stem cell (ED-iPSC) lines from fibroblasts obtained from individuals of African American, Hispanic-Latino, Caucasian and Asian origin to provide an important resource to benefit research and clinical discovery. The significance of our study is that ED-iPSC lines were derived by same viral vector, single method, and same culture conditions throughout the same place, instead of quite diverse iPSCs derivation methods and different individual laboratories. In addition, to optimize comparative analysis of tri-lineage commitment we maintained derived lines under uniform hFGF by using slow release beads. Further, we used lithography templated embryoid bodies of uniform optimized 200 micron size^8^. When we compared our data with previously characterized H9 hESCs, the growth pattern and characterization of ED-iPSCs are similar to hESCs. The availability of these cell lines will be useful to begin to evaluate whether stem cell lines can be used to investigate the contributions of ethnic backgrounds to cell or drug based therapies that have a genetic component to disease onset or resistance, an aspect that is expected to be important as personalized medical procedures advance. Our comprehensive in-depth characterization of these ethnically diverse-iPSC (ED-iPSC) lines derived and characterized under a uniform platform includes karyotype, teratoma analysis, gene expression analysis by qRT-PCR, and *in vitro* analysis under xenofree conditions and using high throughput microarrays to uniformly generate embryoid bodies for optimal comparative evaluation of cell line potential for tri-lineage commitment to differentiation.

## Methods

### *In vitro* culture of primary human fibroblasts and lentivirus reprogramming

Human fibroblasts for iPSCs derivation were obtained from Coriell Institute (Camden, NJ) and reprogrammed using a single polycistronic vector using four-factor 2 A (4F2A) doxycycline (DOX)-inducible lentivirus encoding mouse cDNAs for Oct4, Sox2, Klf4, and c-Myc separated by three different 2 A peptides (P2A, T2A, and E2A, respectively). The lentiviral plasmids are p20321 (TetO-FUW-OSKM) and p20342 (FUW-M2rtTA) (Addgene, Cambridge, MA) originally developed by Carey *et al.*^9^. Lentiviral particles (4F2A and M2rtTA) were packaged in HEK 293T cells. The primary fibroblast cells were co-transfected using the lentivirus construct, psPAX and pCMV-VSVG vectors by calcium phosphate co-precipitation. Viral supernatants from cultures packaging each of the two viruses were pooled, filtered through a 0.45 ◻m filter and concentrated by ultracentrifugation and stored at −80°C.

The 5 human fibroblast lines were transduced by viral particles in xenofree human fibroblast culture medium^10^ in the presence of polybrene (8 μg/mL). Forty-eight hours after infection, less than 15% of fibroblasts tested immunopositive for viral-derived OCT4. The procedure was carried out in 1 well of a 6-well plate with cells at 70% confluence to allow for cell growth after viral infection an appearance of stem cell colonies. The medium was replaced two days after infection, and then daily, with xenofree hES medium plus doxycycline (1 μg/ml) formulated to maintain stem cell pluripotency^10^,^11^,^12^. After 35 days of culture, small cell clumps distinguishable from the fibroblast morphology appeared. Those that formed cell colonies with hESC-like morphology were mechanically isolated and passed on to mitotically inactivated xenofree human foreskin feeder cells (ATCC PCS-201–010). Overall reprogramming efficiency by this method was calculated to be 0.002 ~ 0.004%. The iPSC colonies were expanded for several passages under xenofree conditions without doxycycline and evaluated for expression of markers of pluripotency by quantitative RT-PCR (qRT-PCR) and immunocytology.

### Real-time PCR genomic analysis

Quantitative PCR analysis was done by isolation of total RNA from the hESC or iPSC lines and parental fibroblast lines and purification using the NucleoSpin RNA XS Total RNA isolation kit (Clontech). Reverse transcription (RT) was performed in a 20 ul reaction volume using Superscript II (Invitrogen) and the cDNA reaction was diluted to a 300 ul working stock volume. Primers for use in qPCR were first validated by maximally amplifying cDNA from a range of samples to confirm that a single PCR reaction product was produced and that the amplicon was of the predicted length. For validation, 10 ul of cDNA from H9 hESCs (WA09, Wicell, Madison, WI), control fibroblasts (line A-2), and two of iPSC lines (A-2.2.1 & A-2.2.2) for each primer set was amplified for 36 cycles (95 °C 30 s, 55 °C 30 s, 72 °C 30 s). For endogenous and transgene expression, 5 ul of cDNA from each iPSC lines for each primer set was amplified for 32 cycles and resolved on a 3% nusieve agarose gel and visualized by ethidium bromide staining. Quantitative PCRs contained 10 ng of cDNA, 400 nM of each primer, and SYBR Green PCR Master Mix (AppliedBiosystems). Each sample was analyzed by triplicate by an ABI PRISM 7000 sequence detection system. Data was analyzed using the system’s software. The expression of gene of interest was normalized to GAPDH in all cases and compared with hESCs.

### Mycoplasma analysis of ED-iPSC lines

We used the MycoAlert^TM^ PLUS Assay mycoplasma detection kit (Lonza, Allendale, NJ) essentially as manufacturer’s instructions. Briefly, after centrifugation (1500 rpm, 5 min) of cell supernatant during passage of suspension iPSC cultures, the supernatants were transferred into luminescence compatible tubes (Corning Inc., Corning, NJ). The viable mycoplasma was lysed to allow enzymes to react with MycoAlert^TM^ PLUS substrate, catalyzing the conversion of ADP to ATP. The level of ATP in the sample both before (reading A; ATP background) and after (reading B) the addition of MycoAlert^TM^ PLUS substrate was assessed using a luminometer (Victor^3^, Perkin-Elmer, Waltham, Massachusetts, USA), so that a ratio B/A was obtained. Reading B assesses the conversion of ADP to ATP and is a monitor of contaminated samples. If the ratio of B/A is greater than 1 the cell culture was considered to be contaminated by mycoplasma. For control samples, the MycoAlert TM assay positive and negative control set was used.

### Xenofree stem cell maintenance, passaging in 2D, in 3D as uniform embryoid bodies and directed early differentiation of ED-iPSC and hESC lines

Ethnically diverse-induced pluripotent stem cell (ED-iPSC) lines maintained on human foreskin fibroblast feeders were transferred to feeder-free conditions in non-tissue culture treated dishes coated with xenofree vitronectin (StemCell Technologies, Vancouver, Canada) or 1:100 Matrigel (10 mg/ml; BD Biosciences, San Jose, CA) diluted into Hank’s Buffered Saline Solution (Gibco HBSS; Life Technologies, Grand Island, NY). Cells were maintained in mTeSR2 complete media (StemCell Technologies, Vancouver, Canada) and mechanically passaged between days 5 and 7. Media was replaced on day 1 after the first passage of the series and cells grown overnight. On day 2, slow release Stem Beads® FGF2 (20 microliters of PLGA beads loaded with hFGF2; StemBeads; Stem Culture Inc., Rensselaer, NY) were added with fresh mTeSR2 media. Media changes were done every 3 days with Stem Beads® FGF2 and mTeSR2. Preparation of uniform sized EBs from iPSCs colonies was done in custom lithography template microarrays (LTA) generated in-house. Chemical dissociation of the stem cell colonies into single cell suspension was done before and loading of the cells into LTA- polydimethylsiloxane (PDMS) grids in mTeSR2 media in the presence of 10 μM Rock inhibitor (Sigma-Aldrich, St. Louis, MO) at day 0. Stem cells were maintained in grids for five days with media changes every two days. For directed multi-lineage early differentiation we used the Human Pluripotent Stem Cell Functional Identification Kit (R&D Systems, Minneapolis, MN).

### Immunocytology

For immunocytology of biomarkers in iPSC colonies, cells were prepared by two methods. Cells were fixed using 4% paraformaldehyde in PBS for 15 min at room temperature and blocked by incubating cells for 90 min in a solution containing 3% normal donkey serum and permeabilized by 0.1% Triton-X 100 for 10 min before antibody addition. Incubations with the primary antibodies of anti-Nanog (Santa Cruz Biotechnology, Dallas, TX) and anti-SSEA4 (Santa Cruz Biotechnology, Dallas, TX) were done at 4 °C overnight, followed by incubation with a secondary antibody conjugated with Alexa 647 or Alexa 488 (Abcam, Cambridge, MA). After rinsing with phosphate buffered saline (PBS), the DNA was stained with bisBenzimide H 33258 (Sigma-Aldrich, St. Louis, MO) and cells imaged using a digital camera connected to a Nikon TE-2000 inverted microscope.

Phase imaging for *in vitro* differentiated samples was done on a Nikon 80i epifluorescence microscope using a PLAN 10 × 0.30 NA DL objective and images captured with a cooled QICam CCD camera. Fluorescent images were obtained on a Leica SP5 Laser Scanning Confocal Microscope using either HC PL FLUOTAR 10 × 0.30 NA or HCX PL APO CS 20 X .70 NA objectives and also on a Zeiss AxioObserver Z1 Inverted Microscope with Colibri LED illumination, using a 100 X oil 1.45 NA PlanFLUAR or 63 X Plan-Apochromat 1.4 NA oil DIC objectives. Images were captured with a Hamamatsu ORCA ER CCD camera and Zeiss Axiovision Rel 4.8 acquisition software. Figures were compiled using Adobe Photoshop (Adobe Systems Inc., San Jose, CA) and Microsoft PowerPoint (Microsoft Corp., Redmond, WA) software.

The immunocytology of 2D cell cultures or three dimensional EBs was done by first fixing cells for 10 minutes at room temperature in 4% paraformaldehyde and stored overnight in PBS + 0.1% Tween20 at 4 °C. Immediately before incubation with antibodies, the cells were permeabilized with PBS + 0.5% Triton X-100 for 1 hour at 4 °C. Nonspecific binding was blocked by 20 minute incubation in 1% BSA in HBSS and followed by a single HBSS wash. Antibodies used for gauging pluripotency recognized Oct4A C-10 (Santa Cruz Biotechnology, Dallas, TX) and anti-SSEA4 (Millipore, Billerica, MA) (1:1000 each). Analysis of lineage commitment to differentiation was done using antibodies to OTX2 (ectoderm), SOX17 (endoderm), and Brachyury (mesoderm; 1:100 each) provided in the Human Pluripotent Stem Cell Functional Identification Kit (R&D Systems, Minneapolis, MN). Secondary antibodies were either AlexaFluor 488 or AlexaFluor 594 (A-11001, A-11037, Invitrogen, Carlsbad, CA). Nuclei were stained with bisBenzimide H 33258 (Sigma-Aldrich, St. Louis, MO) at 4 °C overnight and followed by washing one hour in HBSS at 4 °C. Samples were mounted in ProLong Gold antifade reagent (Life Technologies, Grand Island, NY) at 20 °C overnight in the dark before imaging immediately or storing at 4 °C.

### *In vivo* teratoma formation assay

Approximately 2 million ED-iPSCs were injected subcutaneously in the flank region of NOD scid gamma (NSG) mice (The Jackson Lab, Bar harbor, ME). After 12–24 weeks, teratomas were formed from 10 iPSC lines, and tumors were excised & fixed in 10% normal buffered formalin (NBF) overnight. The samples were processed for histology by the Division of Human Pathology at MSU. Hematoxylin- and eosin (H&E)-stained sections were examined under a microscope.

## Additional Information

**How to cite this article**: Ah Chang, E. *et al.* Derivation of Ethnically Diverse Human Induced Pluripotent Stem Cell Lines. *Sci. Rep.* **5**, 15234; doi: 10.1038/srep15234 (2015).

## Figures and Tables

**Table 1:** Ethnically diverse parental fibroblasts and induced Pluripotent Stem Cell Lines.

Sources	Catalogue no	Nature	Sex	Age	Ethnicity	Symbols
Coriell	AG08498	Skin (foreskin)	male	1 year	Asian	A-2
Coriell	AG22284	Skin (foreskin)	male	1 day	Asian	A-3
Coriell	GM00970	Skin (foreskin)	male	26 years	Caucasian	C-1
Coriell	GM22268	Skin (foreskin)	male	1 day	African American	F-3
Coriell	GM22186	Skin (foreskin)	male	1 day	Hispanic Latino	H-3

**Asian**	**Asian**	**Caucasian**	**African American**		**Hispanic Latino**	
A-2.1.1	A-3.1.1	C-1.1.1	F-3.2.2		H-3.1.1	
A-2.2.1	A-3.2.1	C-1.2.1	F-3.3.1		H-3.2.1	
A-2.2.2	A-3.3.1	C-1.3.1	F-3.3.2		H-3.3.1	
A-2.2.3		C-1.5.1	F-3.4.1		H-3.4.1	
A-2.3.1			F-3.4.2		H-3.5.2	
			F-3.5.2		H-3.5.5	
			F-3.6.1			

**Table 2:** Karyotype Results.

iPSC lines	Passagenumber	Karyotype	iPSC lines	PassageNumber	Karyotype
Asian			African-American		
A-2.1.1	p 9	46, XY	F-3.3.2	p 6	46, XY
A-2.2.1	p 12	46, XY	F-3.5.2	p 7	46, XY
A-2.2.2	p 7	46, XY		p 7	46, XY
Asian			Hispanic-Latino		
A-3.1.1	p 11	46, XY	H-3.1.1	p 8	46, XY
A-3.2.1	p 8	46, XY	H-3.3.1	p 9	46, XY
A-3.3.1	p 7	46, XY	H-3.5.2	p 9	46, XY
Caucasian					
C-1.2.1	p 7	46, XY			
C-1.5.1	p 7	46, XY			

A-2.3.1	p 7	46, Y, duo(X) (p22.3q13.1)[20]			
C-1.1.1	p 9	46, XY, r(22)(p13q13.3)/45,XY, -22			
C-1.3.1	p 7	46, XY, r(22)(p12q13)/46,XY[13]			
